# The Brazilian-Portuguese version of the Sleep Hygiene Index (SHI): validity, reliability and association with depressive symptoms and sleep-related outcomes

**DOI:** 10.5935/1984-0063.20190130

**Published:** 2020

**Authors:** André Comiran Tonon, Guilherme Rodriguez Amando, Alicia Carissimi, Juliana Jury Freitas, Nicóli Bertuol Xavier, Guilherme Hidalgo Caumo, Luka Gawlinski Silva, Diogo Onofre Gomes de Souza, Maria Paz Hidalgo

**Affiliations:** 1 Hospital de Clínicas de Porto Alegre, Laboratório de Cronobiologia e Sono - Porto Alegre - RS - Brazil.; 2 Federal University of Rio Grande do Sul, Graduate Program in Psychiatry and Behavioral Sciences - Porto Alegre - RS - Brazil.; 3 Federal University of Rio Grande do Sul, Department of Biochemistry - Porto Alegre - RS - Brazil.; 4 Federal University of Rio Grande do Sul, Department of Psychiatry and Forensic Medicine - Porto Alegre - RS - Brazil.

**Keywords:** Psychiatry, Depression, Sleep Apnea, Sleepiness, Sleep Hygiene

## Abstract

**Objective:**

To translate the Sleep Hygiene Index (SHI) to Brazilian Portuguese, to describe its psychometric properties and to show its association with sleep quality, daytime sleepiness, risk for sleep apnea and depressive symptoms.

**Methods:**

Thirty subjects participated in the cultural adaptation and the item clarity evaluation. Twenty subjects answered the instrument in three different time-points for test-retest reliability. Eighty adult workers completed the SHI, the Pittsburgh Sleep Quality Index (PSQI), the Epworth Sleepiness Scale (ESS), the Beck Depression Inventory (BDI) and the STOP-BANG (S-B).

**Results:**

SHI shows an acceptable internal consistency (Cronbach’s α=0.75), as well as a high reproducibility (intraclass correlation=0.972, p<0.01). The three final factors of confirmatory factor analysis extract an average of 48.22% of the total sample variance. Worse sleep hygiene (higher SHI score) correlated with poor sleep quality (r=0.398, p<0.001), excessive daytime sleepiness (r=0.406, p<0.001) and depressive symptoms (r=0.324, p=0.003). No correlations with S-B were found.

**Conclusions:**

SHI presents satisfactory-to-optimal psychometric properties. This instrument is useful for treatment planning and management of sleep hygiene practices. Thus, it represents a reliable way of assessing sleep hygiene quantitatively in both research and clinical settings.

## INTRODUCTION

Inadequate sleep practices and habits encompass multiple biological and environmental factors, reflecting on sleep quality, sleep duration, unrestful sleep sensation, and excessive daytime sleepiness^1^. Unhealthy lifestyle factors, for instance lack of physical exercise and consumption of substances (i.e., caffeine, alcohol, and nicotine), negatively affect sleep^2^. Moreover, the use of electronic devices such as cellphones, computers, and televisions also have demonstrated to impact sleep quality and duration^3^.

In this scenario, sleep hygiene (SH) is postulated as a set of strategies that incorporate daily adjustments in both behavior and environment aiming to improve sleep^4^. SH recommendations are almost uniformly included as part of cognitive-behavioral treatment programs for insomnia and other sleep disorders^5^. Furthermore, sleep problems are commonly associated with mental health issues, such as depressive mood^6^ and other psychological status^7^, but the exact mechanisms of this bidirectional interaction remain unknown.

There are few validated quantitative instruments designed to evaluate SH. Consequently, it is essential to develop scales that aim to assess SH in order to improve the reliability and reproducibility of studies regarding the topic. Amongst these few, the Sleep Hygiene Index (SHI), developed by Mastin et al.^1^, is a valid and reliable instrument made to assess sleep-related practices and behaviors.

Literature review highlights the necessity for the SHI to be translated to Portuguese, as increasing studies have investigated sleep and sleep-related problems in lusophone nations^8-10^. Therefore, the instrument would contribute to the research and clinical practice of treatment and follow-up of sleep issues^9,10^. Henceforth, this study aims to develop a Portuguese translation and cross-cultural validation of the SHI into Brazilian Portuguese, as well as describe its psychometric properties and its association with sleep quality, daytime sleepiness, risk for sleep apnea and depressive symptoms.

## METHODS

The process of translation, cultural adaptation and establishment of construct validity of the Brazilian Portuguese version of the SHI was based on the recommendations and methodology of Guillemin et al.^11^, de Brito et al.^12^ and Terwee et al.^13^.

### Sample selection and study procedures

The Research Ethics Committee of the Hospital de Clínicas de Porto Alegre approved all procedures (protocol number #2015-0263 GPPG/HCPA). Individuals that accepted participation in the research protocol signed the informed consent after full explanation of study objectives.

For all sample size calculations (i.e., cross-cultural adaptation and clarity, internal consistency, and validity) the previous recommendations by Anthoine et al.^14^ were used. Initially, the process of translation of the original scale was developed, followed by the cultural adaptation and evaluation of clarity of items by primary healthcare professionals (n=12) as well as patients attending family doctor appointments (n=18). With that, the final version of the scale was used for the assessment of test-retest reliability by professionals from primary healthcare settings (n=20). Finally, for the establishment of the Cronbach’s alpha, the construct validity and the correlations of the instrument with depressive symptomatology and sleep questionnaires, adult hospital workers (nurses, nurse assistants, and security staff; n=80) from both sexes were invited to answer study instruments.

### The Sleep Hygiene Index

The Sleep Hygiene Index (SHI) is a self-reported instrument containing 13 items designed to assess behaviors and habits related to maladaptive sleep-related practices^1^. Participants are asked to report how often they engage in such behaviors on a Likert scale (“always”, “frequently”, “sometimes”, “rarely” and “never”). A total continuous score is derived from a sum of all questions, representing a global assessment of sleep hygiene, being higher scores an indicative of poor sleep hygiene (i.e., maladaptive sleep hygiene status). Yet, there is no cut-off established to define groups based on the total score^1,15,16^. This instrument was based on a robust sleep hygiene model in a large nonclinical population.

### Development of the Brazilian Portuguese version of the SHI

Two separate processes of translation were developed. Two groups of native Portuguese speakers with proficient English independently translated the original version of the SHI into Brazilian Portuguese. These translations were assessed by two panels composed of eight scientists with backgrounds in psychology (1), medicine (4), and biomedical science (3). This revision aimed to identify translation errors and to certify content validity. The revised drafts were translated back into English by two independent translators who are fluent in Brazilian Portuguese and are English native speakers. The translators had not been informed about the original scale or about the study objectives. The same revision teams from the previous step compared the back-translation with the original instrument in English. The back-translation process was used to find and correct any errors in the Portuguese draft that would have led to inconsistencies with the original version.

A conciliatory version was determined by analyzing both translated versions. For this version, the research team considered the Brazilian linguistic and cultural context, aiming to achieve an instrument that was accessible to individuals with different social backgrounds, also preserving the rationale of the original scale. The conciliatory version underwent the following steps: cultural adaptation, evaluation of the clarity of items, analysis of reliability and reproducibility, and construct validity. The process from the original translation to the final conciliatory version is available in the Supplementary File 1, available as an appendix.

### Cultural adaptation and evaluation of the clarity of items

The conciliatory version was given to graduate students and individuals with higher education (n=12) and patients from the primary healthcare setting (n=18) for the assessment of clarity and adjustment to different cultural backgrounds. These participants evaluated the clarity of all items based on a visual analog scale (VAS; Table 1) ranging from “not at all clear/comprehensible” to “very clear/comprehensible”. The participants were also given the opportunity to provide further feedback and suggestions on how to adapt the instrument. These remarks were taken and reviewed by the research team, in order to achieve the final consensus version of the SHI in Brazilian Portuguese (see Supplementary Table S1, available as an appendix).

**Table 1:** Psychometric properties and content validity of the Brazilian Portuguese version of the SHI.

SHI items	Internal consistency and reliability			International recommendations^1^			
	Item clarity (VAS from 0 to 10)	Cronbach's alpha if item is deleted	Intraclass correlation (95% CI)	SNF	AAHS	HS-HMS	ASA
Cronbach's alpha = 0.752							
1. Daytime naps	9.83	0.747	0.908 (0.807-0.961)	x		x	x
2. Regular bedtime	9.62	0.741	0.949 (0.889-0.979)	x	x	x	x
3. Regular get-up time	9.22	0.746	0.861 (0.707-0.941)		x	x	x
4. Nighttime physical exercise	9.79	0.767	0.859 (0.706-0.939)			x	x
5. Prolonged time in bed	9.64	0.744	0.907 (0.806-0.960)				
6. Use of stimulants close do bedtime	9.90	0.734	0.966 (0.930-0.986)	x	x	x	x
7. Doing activities that promote wakefulness prior to sleeping	9.66	0.722	0.919 (0.831-0.965)	x	x	x	x
8. Distressed emotional states at bedtime	9.75	0.719	0.899 (0.789-0.957)		x	x	x
9. Use of bed for activities other than sleeping or sex	9.85	0.728	0.979 (0.957-0.991)		x	x	x
10. Inadequate bed conditions	9.88	0.744	0.786 (0.545-0.909)	x	x		x
11. Inadequate room conditions (e.g. light, temperature and noise)	9.87	0.738	0.941 (0.876-0.975)	x	x		x
12. Dealing with important matter at bedtime	9.84	0.724	0.931 (0.857-0.971)	x		x	x
13. Worrying in bed/ nervousness in bed	9.88	0.715	0.911 (0.812-0.962)	x		x	x

1 For each Sleep Hygiene Index item, an "x" represents the presence of sleep hygiene advice in the recommendations of these four international societies. Clarity items are represented as mean values of all responses.  AAHS=American Alliance for Healthy Sleep; ASA=American Sleep Association; HS-HMS=Healthy Sleep - Harvard Medical School; SNF=Sleep National Foundation; VAS=Visual-analog scale.

### Internal consistency and reproducibility

The Cronbach’s alpha was used as an internal consistency estimate. Furthermore, 20 individuals answered the instrument in three different time points: baseline, three hours later, and two weeks later. This process, called test-retest reliability, examines the reproducibility of the self-reports of the SHI in different moments, without any intervention with the tested subjects.

### Validity

Face validity is defined as the capacity of an instrument to assess what it was designed to measure. In this study, face validity was determined by the multidisciplinary committee responsible for the Brazilian version of the SHI.

Content validity is defined as the rate to which either item applies to measure the target content. The content of the Brazilian Portuguese SHI is entirely based on the original validated instrument. Still, in order to establish the content validity of the content, each item of the SHI was compared to the SH recommendations of four different international societies (e.g. American Alliance for Healthy Sleep^17^, American Sleep Association^18^, Healthy Sleep - Harvard Medical School^19^, Sleep National Foundation^20^).

Construct validity is the process by which the correlation of a measure with other variables is tested, aiming to analyze theoretical consistency. Considering previous evidence on the associations of SH with sleep-related outcomes^1,12,16^, this study’s hypothesis is that sleep quality and daytime sleepiness associate with SH. In addition, the SHI was correlated with risk for sleep apnea, expecting no significant associations, as this latter assessment is mainly based on constitutional variables (e.g., body mass index, age, gender, neck circumference), which are not expected to vary according to the modification of sleep-related habits. Finally, previous studies report that sleep hygiene practices seem to be associated with depressive symptomatology^6,7^. Therefore, we expanded our correlation analyses aiming to verify this association with the Brazilian Portuguese version of the SHI.

The construct validity was also tested using exploratory factor analysis.

## INSTRUMENTS

### Sleep quality

The Pittsburgh Sleep Quality Index (PSQI) is a self-reported questionnaire that assesses sleep quality over the past month^21^. The PSQI contains 19 items and seven components (i.e., sleep duration, sleep latency, habitual sleep efficiency, sleep disturbances, daytime disturbance, subjective sleep quality, and use of sleep-promoting substances). For this study, the validated Brazilian Portuguese version of the PSQI was used^22^.

### Daytime sleepiness

The Epworth Sleepiness Scale (ESS) is a questionnaire designed for subjective assessment of daytime sleepiness^23^. Individuals are asked to answer their likelihood to fall asleep in 8 daily hypothesized situations. For this study, the validated Brazilian Portuguese version of ESS was used^24^.

### Sleep apnea

The STOP-BANG (S-B) is a self-reported questionnaire that measures the risk of obstructive sleep apnea (OSA)^25^. This questionnaire consists of 8 dichotomous items in a yes/no structure. The version used in this study is a validated version for Brazilian-Portuguese populations^26^.

### Depressive symptoms

The Beck Depression Inventory (BDI) is self-reported 21-item inventory that assesses symptoms and attitudes related to depression symptomatology^27^. For this work, the validated Brazilian Portuguese version was used^28^.

### Statistical Analyses

The Shapiro-Wilk test assessed normality in data distribution. Student’s t test for independent samples was used for group comparisons of parametric data. Intraclass correlation analyses were used for test-retest reliability, aiming to compare items and total score within the timepoints. For the exploratory factorial analyses, the Varimax rotation method extracted principal components and the number of factors suggested to be retained was based on Kaiser criteria (Eigenvalues > 1) and the scree plot. Pearson’s correlation analyses compared the continuous SHI score with other study instruments. The values for Pearson’s correlation were considered: weak (0 - 0.3), moderate (0.3 - 0.7), strong (0.7 - 0.9) and very strong (0.9 - 1).

The analyses were performed using SPSS for Windows (version 19; SPSS Inc., Chicago, IL) and all graphs were generated using GraphPad Prism version 7.0 for Windows (GraphPad Software, San Diego, CA). Values of *p*<0.05 were considered statistically significant.

## RESULTS

The presented version of the Sleep Hygiene Index (SHI) showed no significant changes in the structure of the questionnaire according to the translation process into Brazilian Portuguese. The main alterations were linguistic adaptations that aimed to guarantee the adequate cross-cultural validation of the instrument.

The final version of the Brazilian-Portuguese SHI (see Supplementary File 1, available as an appendix) was filled by eighty hospital workers (descriptive statistics shown in Table 2). The scale shows an acceptable internal consistency, as measured by a Cronbach’s α of 0.75, as well as a high reproducibility estimate for the total score (intraclass correlation=0.972, 95% confidence interval=0.941-0.988, *p*<0.01).

**Table 2:** Descriptive statistics.

	Female (n=37)	Male (n=43)	Total (n=80)
Age	44.78±8.77	44.74±7.29	44.76±7.95
Sleep quality (PSQI)	7.35±4.33	6.81±3.39	7.06±3.84
Daytime sleepiness (ESS)	11.08±5.2	9±5.1	9.96±5.23
Risk for OSA (S-B)	1.92±1.91	3.79±1.58	2.93±1.97
Depressive symptoms (BDI)	9.32±6.37	8.03±6.23	8.04±6.24
Sleep hygiene (SHI)	30.84±5.58	29.42±7.75	30.08±8.83

BDI=Beck Depression Inventory; ESS=Epworth Sleepiness Scale; PSQI=Pittsburgh Sleep Quality Index; S-B=STOP-BANG; SHI=Sleep Hygiene Index.

The construct validity of the Portuguese SHI version was tested using exploratory factor analyses. The Kaiser-Meyer-Olkin measure of sampling adequacy was 0.71. The Bartlett’s chi-square value (214.76; *p*<0.001) indicates the appropriateness of the data for the analyses. The Kaiser criteria (Eigenvalues > 1) and the scree plot suggested five factors to be retained. However, according to the theoretical sense of the items in each of the five factors, the model that provided the most desirable rotated factor structure was the three-factor model. Thus, a confirmatory analysis was conducted with the three defined factors (Table 3). The three factors extract an average of 48.22% of the total sample variance (F1=26.80%, F2=11.67% and F3=9.76%), that explained a variance for each factor to the total variance among all SHI questions.

**Table 3:** Factor loadings for the Sleep Hygiene Index (SHI) items.

SHI items	Factor 1	Factor 2	Factor 3
	Sleep disturbing behavior and environment	Bedtime proceedings	Irregular sleep-wake schedule
1. Daytime naps	0.027	0.641	0.058
2. Regular bedtime	0.203	0.054	0.749
3. Regular get-up time	-0.042	0.222	0.828
4. Nighttime physical exercise	-0.120	0.241	0.094
5. Prolonged time in bed	0.078	0.705	0.114
6. Use of stimulants close do bedtime	0.424	0.082	0.408
7. Doing activities that promote wakefulness prior to sleeping	0.649	0.031	0.202
8. Distressed emotional states at bedtime	0.678	0.265	0.037
9. Use of bed for activities other than sleeping or sex	0.453	0.584	-0.078
10. Inadequate bed conditions	0.694	-0.217	-0.115
11. Inadequate room conditions	0.549	-0.165	0.298
12. Dealing with important matter at bedtime	0.671	0.113	0.079
13. Worrying in bed/ nervousness in bed	0.583	0.486	0.105
Eigenvalues	3.48	1.52	1.27
% of variance	0.27	0.12	0.10

**Figure 1:**
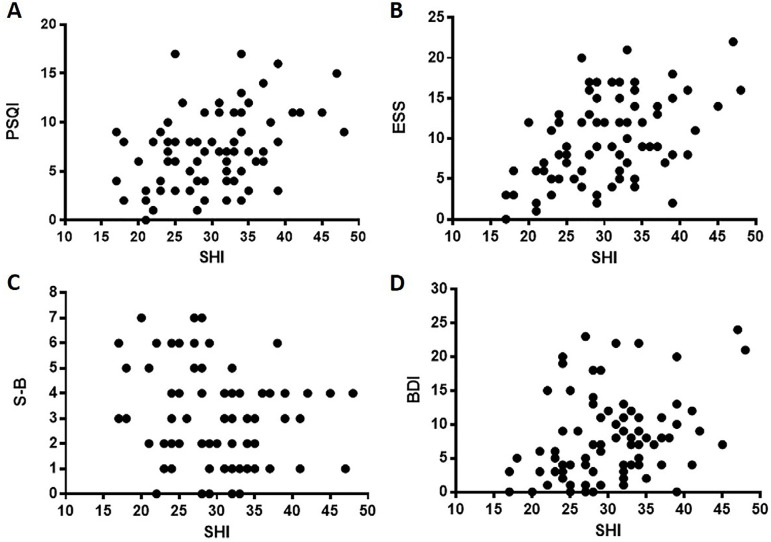
Correlations of sleep hygiene scores (Sleep Hygiene Index, SHI) with sleep quality (Pittsburgh Sleep Quality Index, PSQI, A), daytime sleepiness (Epworth Sleepiness Scale, ESS, B), risk for sleep apnea (STOP-BANG, S-B, C), and depressive symptoms (Beck Depression Inventory, BDI, D). Values are for Pearson’s correlation coefficient and significance levels.

The final SHI score positively correlated with total PSQI (r=0.398, *p*<0.001), ESS (r=0.406, *p*<0.001) and BDI (r=0.324, *p*=0.003) scores (Figure 1). No significant associations were found between the SHI final score and the risk for sleep apnea measured by S-B. Sex differences were only significant for S-B data (t =-4.8, *p*<0.001).

## DISCUSSION

The Brazilian Portuguese version of the Sleep Hygiene Index (SHI) is an easy-to-use, self-administered instrument that can complement the evaluation of sleep issues both in clinical and research settings. This study used international recommendations and based its methods on previous high-quality studies to ensure that the Brazilian Portuguese translation of the SHI were both reliable and appropriate to the cultural context. The SHI is based on a robust SH model tested for its original version. Hence, in this study, the instrument was compared to the SH practices recommended by four different international societies, corroborating its content validity.

Individuals that were asked to answer the scale on every step of the methodology found the scale easy to comprehend. This quality is also reflected in the high levels of reported clarity of all items (Table 1). The instrument shows acceptable internal consistency (α=0.75), which is higher than the original English version (α=0.66)^1^. Similarly, a satisfactory internal consistency was found in a nonclinical sample of Nigerian students (α=0.64)^16^ and in a Korean sample with chronic pain (α=0.75)^15^. Even though sleep habits constantly vary in an individual’s life course, this study shows a high reproducibility for the Brazilian version of the SHI, as measured by intraclass correlations.

The construct validity of the Portuguese SHI version was tested using exploratory factor analyses, and three factors were obtained. The first factor in this Brazilian sample, defined as “sleep disturbing behavior and environment,” was composed by most items. The second factor was defined as “bedtime proceedings.” Resembling the results from a sample of Nigerian undergraduate students^16^, the items “regular bedtime” and “regular get-up time” were grouped in the third factor, defined as “Irregular sleep-wake schedule.” For both samples, this factor demonstrates a high value for each item loaded. Additionally, Chehri et al.^29^ and Ozdemir et al.^30^ found that the items loaded differently in the general Persian population and Turkish clinical and non-clinical samples, respectively. This difference can be attributed to cultural differences and sample characteristics.

The average score for the SHI is slightly lower compared to the one found in the original scale (30.02±6.82 in this study compared to 34.66±6.6 in the original^1^), with no significant sex differences. This study confirms the hypothesis that SH is associated to sleep quality and daytime sleepiness, but not to the risk of sleep apnea. The moderate correlations of the SHI with the PSQI and the ESS endorse the construct validity of the presented instrument. These results also point to the relevance of including assessments of SH in studies regarding sleep issues. Indeed, available evidence suggest that SH counseling^4^ including sleep time regularity, avoidance stimulants beverage and daytime napping, improve sleep quality^7, 31^ and reduce daytime sleepiness^30,32^. Sleep apnea is a medical condition commonly associated to constitutional factors that are not expected to change with better sleep practices. Thus, the absence of a significant correlation strengthens the construct validity of the SHI. Furthermore, a moderate correlation between inadequate sleep hygiene and depressive symptoms was observed. This relationship is in line with recent reports indicating that sleep hygiene strategies are somewhat related to depressive symptomatology^6,30,33,34^.

A homogeneous convenience sample size of hospital workers was chosen to guarantee internal consistency to our findings. However, this may be a limitation of this study because 1) a convenience sample represents a risk for selection bias and 2) we only studied hospital workers from a community in south Brazil and it is not possible to control for socio-cultural and work-related aspects of this population. Moreover, our study was primarily based on self-reported measurements, and no diagnostic interview was performed. Nevertheless, the results are similar to other studies that assessed SH using the SHI^1,16,30^. In addition, even though we calculated our sample size *a priori*, it might have underestimated the correlation analyses. Future studies would highly contribute to the topic by exploring clinical samples and different settings.

## CONCLUSIONS

The SHI is a simple self-report measure which presents satisfactory-to-optimal psychometric properties. This report shows moderate significant correlations of inadequate sleep hygiene with poor sleep quality, daytime sleepiness and depressive symptoms in an adult nonclinical population. This instrument can be useful in the treatment planning and in the management of sleep hygiene practices. Thus, it represents a feasible and reliable way of assessing sleep hygiene quantitatively in both research and clinical settings.

## ONLINE-ONLY SUPPLEMENTARY MATERIAL

**Supplementary File 1:**
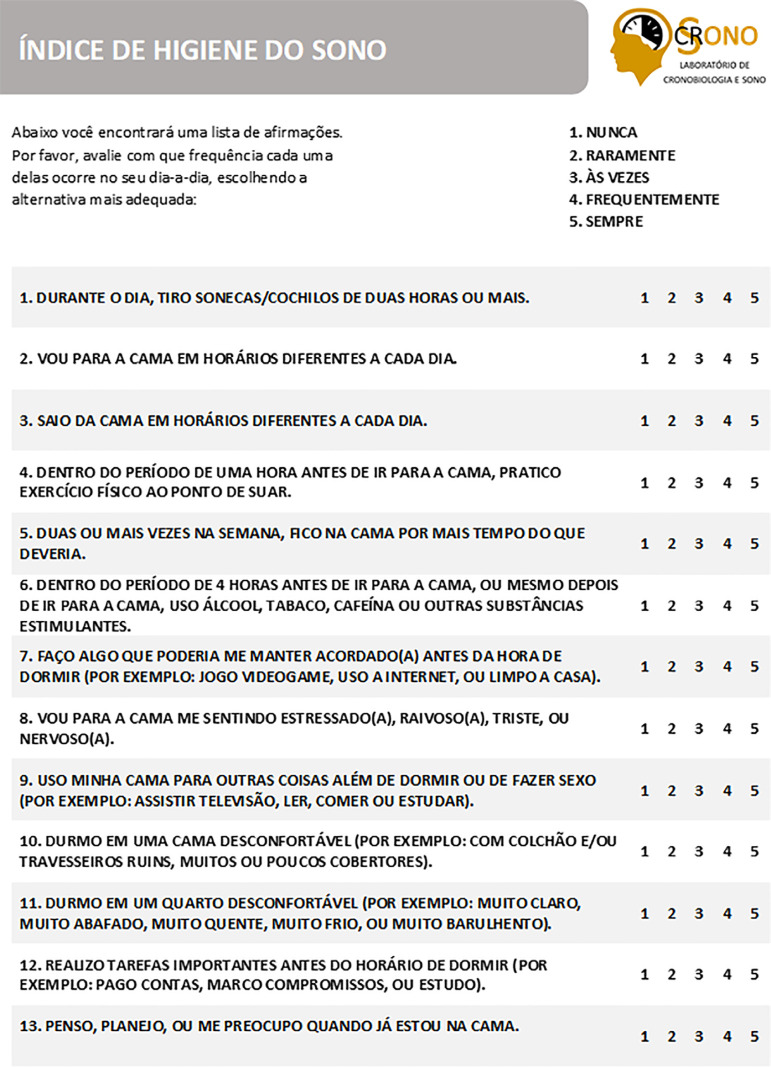
Índice de Higiene do Sono, Brazilian-Portuguese Version of the Sleep Hygiene Index (SHI).

Hospital de Clínicas de Porto Alegre (HCPA)

Universidade Federal do Rio Grande do Sul (UFRGS)

Para contato e informações sobre a escala: labcronoesono@hcpa.edu.br

**Table S1:** Process of Translation and Cross-cultural Adaptation of the Brazilian-Portuguese Version of the Sleep Hygiene Index (SHI)

**ORIGINAL**
Below you will find a list of statements. Please rate how true each statement is for you by circling a number next to it. Use the scale to make your choice.
0 Never
1 Rarely
2 Sometimes
3 Frequent
4 Always

**Sleep Hygiene Index Items**
1. I take daytime naps lasting two or more hours.
2. I go to bed at different times from day to day.
3. I get out of bed at different times from day to day.
4. I exercise to the point of sweating within 1 h of going to bed.
5. I stay in bed longer than I should two or three times a week.
6. I use alcohol, tobacco, or caffeine within 4 h of going to bed or after going to bed.
7. I do something that may wake me up before bedtime (for example: play video games, use the internet, or clean).
8. I go to bed feeling stressed, angry, upset, or nervous.
9. I use my bed for things other than sleeping or sex (for example: watch television, read, eat, or study).
10. I sleep on an uncomfortable bed (for example: poor mattress or pillow, too much or not enough blankets).
11. I sleep in an uncomfortable bedroom (for example: too bright, too stuffy, too hot, too cold, or too noisy).
12. I do important work before bedtime (for example: pay bills, schedule, or study).
13. I think, plan, or worry when I am in bed

**TRANSLATION v1**
Abaixo você encontrará uma lista de afirmações. Por favor avalie a veracidade de cada afirmação para você por circular um número próximo a tal. Use a escala para fazer sua escolha.
0 Nunca
1 Raramente
2 Às vezes
3 Frequentemente
4 Sempre

**Itens de Índice de Higiene de Sono:**
1. Tomo sonecas durante o dia que duram aproximadamente duas ou mais horas.
2. Vou para a cama em diferentes horas no dia a dia.
3. Saio da cama em diferentes horas no dia a dia.
4. Me exercito ao ponto de suar dentre uma hora antes de ir para a cama.
5. Fico na cama por mais tempo de o que deveria duas ou três vezes na semana.
6. Uso álcool, tabaco, ou cafeína dentre quatro horas antes de ir para a cama ou depois de ir para a cama.
7. Faço algo que poderia me acordar antes da hora de dormir (por exemplo: jogar jogos de vídeo game, usar a internet, ou lavar).
8. Vou para a cama me sentindo estressado/a, irritado/a, triste, ou nervoso/a.
9. Uso minha cama para outras funções além de dormir ou sexo (por exemplo: assistir televisão, ler, comer, ou estudar).
10. Durmo em uma cama desconfortável (por exemplo: um colchão ou travesseiro ruim, muitos ou poucos lençóis).
11. Durmo em um quarto desconfortável (por exemplo: muito claro, muito abafado, muito quente, muito frio, ou muito barulhento).
12. Faço trabalho importante antes do horário de dormir (por exemplo: pagar contas, agendar, ou estudar).
13. Penso, planejo, ou me preocupo quando estou na cama.

**TRANSLATION v2**
Abaixo você encontrará uma lista de afirmações. Por favor, avalie o quão verdadeira cada afirmação é para você circulando um número ao lado dela que corresponda à seguinte escala:
0 Nunca
1 Raramente
2 Às vezes
3 Frequentemente
4 Sempre

**Itens do Índice de Higiene do Sono:**
1. Eu tiro sonecas durante o dia que duram duas horas ou mais.
2. O horário em que me deito para dormir varia a cada dia
3. O horário em que me levanto da cama varia a cada dia.
4. Eu realizo exercícios físicos até suar durante a última hora antes de me deitar.
5. Eu fico na cama mais tempo do que deveria duas ou três vezes por semana.
6. Eu uso álcool, tabaco ou cafeína dentro de 4 horas antes ou depois de me deitar.
7. Eu realizo alguma atividade que pode me deixar desperto antes de dormir (por exemplo: jogo videogame, uso a internet ou faço limpeza).
8. Eu me deito para dormir sentindo-me estressado, irritado, chateado ou nervoso.
9. Eu uso minha cama para outras coisas além de dormir ou fazer sexo (por exemplo: assistir televisão, ler, comer ou estudar).
10. Eu durmo em uma cama desconfortável (por exemplo: colchão ou travesseiro ruins, muitas ou poucas cobertas).
11. Eu durmo em um quarto desconfortável (por exemplo: muito claro, muito abafado, muito quente, muito frio ou muito barulhento).
12. Eu realizo tarefas importantes antes de dormir (por exemplo: pago contas, faço planejamento ou estudo).
13. Eu penso, planejo, ou me preocupo quando já estou na cama.

**BACKTRANSLATION v1**
Below you will encounter a list of statements. Please evaluate each statement's veracity to you by circling the number closest to it. Use the scale to make your choice.
0 Never
1 Rarely
2 Sometimes
3 Frequently
4 Always

**Sleep Hygiene Index Items**
1. I take naps over the day that last approximately two or more hours.
2. I go to bed at varying times from day to day.
3. I get out of bed at varying times from day to day.
4. I exercise to the point of sweating within an hour before going to bed.
5. I spend more time in bed than I should two or three times a week.
6. I use alcohol, tabacco or caffeine within four hours before going to bed for after going to bed.
7. I do something that could wake me up before going to sleep (for example: playing video games, using the internet, or washing).
8. I go to bed feelings tressed, irritated, sad or nervous.
9. I use my bed for functions other than sleep or sex (for example: watching television, reading, eating or studying).
10. I sleep on an uncomfortable bed (for example: a bad matress or pillow, too many or too few sheets).
11. I sleep in an uncomfortable room (for example: too bright, too stuffy, too hot, too cold or too noisy).
12. I do important work before going to sleep (for example: paying bills, making appointments, or studying).
13. I think, plan and worry while I'm in bed.

**BACKTRANSLATION v2**
Below you will encounter a list of statements. Please rate how true each statement is to you by circling the number closest to it. Use the scale to make your choice.
0 Never
1 Rarely
2 Sometimes
3 Frequently
4 Always

**Sleep Hygiene Index Items**
1. I take naps during the day that last two hours or more.
2. The time I go to sleep varies from day to day.
3. The time I get out of bed varies from day to day.
4. I exercise until I sweat within an hour of going to bed.
5. I oversleep [stay in bed longer than I should] two or three times a week.
6. I use alcohol, tobacco or caffeine within four hours of going to bed or waking up.
7. I perform actives that can keep me awake before bed (Examples: playing video games, surfing the internet or cleaning.)
8. I go to bed feeling stressed, angry, upset or anxious.
9. I use my bed for things other than sleeping or sex. (Examples: watching TV, reading, eating or studying.)
10. I sleep in an uncomfortable bed. (Example: bad mattress or pillow, too many or too few blankets.)
11. I sleep in an uncomfortable room. (Example: too bright, too stuffy, too hot or cold, or too noisy.)
12. I perform important tasks before bed. (Example: pay bills, make future plans or do schoolwork.)
13. I think, make plans, or worry about things as I am lying in bed.

**CONCILIATORY VERSION**
Abaixo você encontrará uma lista de afirmações. Por favor, avalie com que frequência cada uma delas ocorre no seu dia-a-dia, escolhendo a alternativa mais adequada.
0 Nunca
1 Raramente
2 Às vezes
3 Frequentemente
4 Sempre

**Itens do Índice de Higiene do Sono:**
1. Durante o dia, tiro sonecas de duas ou mais horas.
2. Vou para a cama em horários diferentes a cada dia.
3. Saio da cama em horários diferentes a cada dia.
4. Dentro do período de uma hora antes de ir para a cama, pratico exercício físico ao ponto de suar.
5. Duas ou mais vezes na semana, fico na cama por mais tempo do que deveria.
6. Dentro do período de 4h antes de ir para a cama, ou mesmo depois de ir para a cama, uso álcool, tabaco, cafeína ou outras substâncias estimulantes.
7. Faço algo que poderia me manter desperto/a antes da hora de dormir (por exemplo: jogar videogame, usar a internet, ou limpar a casa).
8. Vou para a cama me sentindo estressado/a, raivoso/a, triste, ou nervoso/a.
9. Uso minha cama para outras coisas além de dormir ou de fazer sexo (por exemplo: assistir televisão, ler, comer, ou estudar).
10. Durmo em uma cama desconfortável (por exemplo: com colchão e/ou travesseiros ruins, muitos ou poucos cobertores).
11. Durmo em um quarto desconfortável (por exemplo: muito claro, muito abafado, muito quente, muito frio, ou muito barulhento).
12. Lido com assuntos importantes antes do horário de dormir (por exemplo: pagar contas, marcar compromissos, ou estudar).
13. Fico pensando, planejando, ou me preocupando quando já estou na cama.

**"ÍNDICE DE HIGIENE DO SONO" - FINAL VERSION**
Abaixo você encontrará uma lista de afirmações. Por favor, avalie com que frequência cada uma delas ocorre no seu dia-a-dia, escolhendo a alternativa mais adequada.
0 Nunca
1 Raramente
2 Às vezes
3 Frequentemente
4 Sempre
**Itens do Índice de Higiene do Sono:**
1. Durante o dia, tiro **sonecas/cochilos** de duas ou mais horas.
2. Vou para a cama em horários diferentes a cada dia.
3. Saio da cama em horários diferentes a cada dia.
4. Dentro do período de uma hora antes de ir para a cama, pratico exercício físico a ponto de suar.
5. Duas ou mais vezes na semana, fico na cama por mais tempo do que deveria.
6. Dentro do período de 4h antes de ir para a cama, ou mesmo depois de ir para a cama, uso álcool, tabaco, cafeína ou outras substâncias estimulantes.
7. Faço algo que poderia me manter **acordado/a** antes da hora de dormir (por exemplo: **jogo** videogame, uso a internet, ou **limpo** a casa).
8. Vou para a cama me sentindo estressado/a, raivoso/a, triste, ou nervoso/a.
9. Uso minha cama para outras coisas além de dormir ou de fazer sexo (por exemplo: assistir televisão, ler, comer, ou estudar).
10. Durmo em uma cama desconfortável (por exemplo: com colchão e/ou travesseiros ruins, muitos ou poucos cobertores).
11. Durmo em um quarto desconfortável (por exemplo: muito claro, muito abafado, muito quente, muito frio, ou muito barulhento).
12. **Realizo tarefas** importantes antes do horário de dormir (por exemplo: **pago**contas, **marco** compromissos, ou **estudo**).
13. Fico pensando, planejando, ou me preocupando quando já estou na cama.
